# Association Between Distance to the Transplant Center and Survival Following Living Donor Liver Transplantation

**DOI:** 10.1002/ags3.70051

**Published:** 2025-06-09

**Authors:** Hajime Matsushima, Akihiko Soyama, Takanobu Hara, Ayaka Kinoshita, Takashi Hamada, Kazushige Migita, Ayaka Satoh, Hajime Imamura, Tomohiko Adachi, Susumu Eguchi

**Affiliations:** ^1^ Department of Surgery Nagasaki University Graduate School of Biomedical Sciences Nagasaki Japan

**Keywords:** graft survival, liver transplantation, living donors, survival access to care

## Abstract

**Aim:**

To determine whether the distance from home to our transplant center affects posttransplant survival in patients undergoing living donor liver transplantation.

**Methods:**

Data from 301 adult patients who underwent primary living donor liver transplantation at our center between January 2000 and July 2023 were retrospectively reviewed. The patients were divided into three groups according to the distance of their homes from our center: Group 1, 0–9 miles (*n* = 104); Group 2, 10–40 miles (*n* = 121); and Group 3, > 40 miles (*n* = 76).

**Results:**

Graft and patient survival rates were significantly lower in Group 3 than in Group 1 (*p* = 0.010 and *p* = 0.004, respectively). Multivariate analysis showed that living > 40 miles from our transplant center was independently associated with worse patient survival (*p* = 0.025). Furthermore, conditional survival analysis revealed that living > 40 miles from our transplant center was associated with impaired 3‐year patient survival. Subgroup analysis of Group 3 revealed that patients living > 40 miles to the nearest transplant center or university hospital had significantly lower 3‐year conditional survival rates than those of patients living < 40 miles from the nearest facility (*p* = 0.011).

**Conclusion:**

Greater distances to the transplant center may negatively affect posttransplant outcomes. Therefore, patients who have undergone living donor liver transplantation and who reside in areas far from specialized medical services should be monitored with caution.

## Graphical abstract

This study investigated the impacts of distance to transplant center on posttransplant survival following LDLT. Patients living further away from the transplant center had worse graft and patient survival rates than those of patients living closer, probably due to less frequency of follow‐up visits, and living > 40 miles from our treatment center was independently associated with worse patient survival. Our findings highlight the importance of seamless follow‐up by specialized medical service.

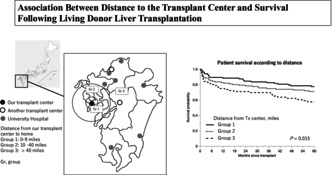

## 1 Introduction

Liver transplantation (LT), a life‐saving treatment for patients with liver failure and hepatocellular carcinoma [1], requires surgical expertise and specialized medical care, and is therefore typically performed in specific transplant centers. As there are relatively few of these centers, patients may need to travel long distances to undergo LT and follow‐up.

Studies from the United States have reported that patients with cirrhosis living at greater distances from transplant centers experience worse outcomes, with a lower likelihood of undergoing deceased donor LT (DDLT) leading to increased waitlist mortality [2, 3, 4]. These findings have been validated by a national registry analysis performed in the United Kingdom [5], and similar results have been reported for the transplantation of other solid organs [6, 7]. In the United States, the allocation of livers from deceased donors has been amended to ameliorate these geographic barriers [8, 9, 10]. Research on the impact of the distance from home to the transplant center has mainly focused on waitlist mortality in DDLT. However, this distance may also influence transplant outcomes because recipients require specialized medical care, including accurate immunosuppressive therapy, precautionary treatments for infectious diseases, and monitoring of allograft function. Although a previous study reported that distance to the transplant center was not associated with one‐year posttransplant survival following DDLT [11], the effects of distance on long‐term survival have not been fully assessed. To date, only one study has reported long‐term outcomes following LT and showed no effect of medical accessibility on outcomes [12]. However, the study involved patients who had undergone DDLT; studies on the influence of distance to the transplant center on long‐term survival following living donor LT (LDLT) are lacking.

In this study, we sought to determine whether the distance to our center affected posttransplant outcomes in patients who had undergone LDLT. Considering that patients could visit a closer transplant center or specialized medical service for rapid assessment and treatment during the posttransplant period, we also investigated the association between the distance to the nearest specialized medical service and posttransplant outcomes in patients living far from our center.

## 2 Methods

### 2.1 Study Population

This retrospective cohort study included consecutive adult patients (aged ≥ 18 years) who underwent LDLT between January 2000 and July 2023 at Nagasaki University Hospital, identified using a prospectively maintained database. This study was approved by the Ethics Committee of Nagasaki University Hospital (Approval No. 20012022‐2) and conducted in accordance with the principles outlined in the Declaration of Helsinki. The requirement for informed consent was waived owing to the retrospective nature of the study. Patients residing outside Japan and those undergoing retransplantation were excluded from the analyses.

### 2.2 Data Collection

Data were collected on demographic and clinical characteristics, including sex, age, body mass index (BMI), etiology of liver disease, Model for End‐Stage Liver Disease (MELD) score at the time of LT, acute‐on‐chronic liver failure (ACLF), pretransplant medical condition, presence of portal vein thrombosis, donor age, ABO incompatibility, graft type, and graft‐to‐recipient weight ratio (GRWR). Data were also collected on operative factors, including operative time, blood loss, blood transfusion, and warm and cold ischemia times. ACLF was diagnosed according to previously described criteria [13]. The protocols for graft selection, splenectomy during LDLT, and immunosuppressive therapy at our center have been described previously [14, 15].

Discharge decisions were made based on stable liver function with appropriate dosage of immunosuppressants, absence of unresolved surgical complications requiring further interventions, sufficient oral intake, and unnecessity of intravenous fluids. Even when liver function was stable but oral intake was not yet considered sufficient or further rehabilitation was still required, the patients were transferred to another facility. In general, we recommended the patients who lived far from our center be transferred to another nearby hospital before being discharged to their homes. At the time of discharge, to evaluate graft function, laboratory data including aspartate aminotransferase (AST), alanine aminotransferase (ALT), bilirubin, and international normalized ratio (INR) were assessed in patients without in‐hospital mortality.

After LDLT, patients with stable graft function were typically followed up every 2–3 months. Patients were advised to contact the transplant center if they experienced symptoms, such as fever, fatigue, appetite loss, abdominal pain, and dyspnea. Depending on the severity of the symptoms, they were then asked to visit either our center or a nearby specialized medical service. To investigate the situation of long‐term follow‐up at our center, the actual numbers of visits to our center per year after 3 years since LDLT were determined in each patient.

### 2.3 Distance Calculation

Travel distance was defined as the linear distance from the residential address of the patient at the time of LT to our center and was calculated using Google Maps. Patients were divided into the following three categories based on distance to our center: Group 1, 0–9 miles, approximating to less than 1 h of travel; Group 2, 10–40 miles, approximating to 1–2 h of travel; and Group 3, > 40 miles, approximating to more than 2 h of travel. The distance from the residential address of the patient to the nearest transplant center or university hospital that could provide subspecialty care was also calculated in the same way.

### 2.4 Data Analysis

To investigate the effects of distance to our transplant center on posttransplant outcomes, patient and graft survival were compared between the groups. Surgical outcomes, including vascular complications, biliary complications, acute rejection, the incidences of early allograft dysfunction (EAD) and small‐for‐size syndrome (SFSS), and 90‐day mortality rates were also compared. The causes of death were also investigated. EAD was defined as previously described [16]. SFSS was determined as described by Dahm et al. [17] and Soejima et al. [18].

### 2.5 Statistical Analysis

Continuous variables are expressed as median and interquartile range, whereas categorical variables are expressed as number and percentage. Categorical variables were compared using the Pearson's chi‐squared test or Fisher's exact tests as appropriate. Continuous variables were compared using the Wilcoxon rank‐sum test. Overall graft and patient survival rates were estimated using the Kaplan–Meier method and analyzed using log‐rank tests. Conditional survival analyses were performed as previously described [19]. The Kaplan–Meier method and log‐rank tests were performed among the patients who have survived over a certain time period. For instance, in 3‐year conditional survival analysis, the patients who had survived over 3 years after LDLT were evaluated. Risk factors associated with patient survival were identified using multivariate Cox regression analysis. To further confirm the prognostic value of distance, 1:1 propensity score matching (PSM) was performed using a caliper of 0.20; survival outcomes were compared between patients living at distances of less or more than 40 miles. Propensity scores were calculated on the basis of the following factors: recipient age, primary liver disease (hepatocellular carcinoma and hepatitis C), MELD score, ACLF, medical condition at the time of LT, ABO incompatibility, graft type, donor age, and the decade the transplant was performed in. All statistical analyses were performed using JMP Pro software version 16 (SAS Institute, Cary, NC, USA). *p* < 0.05 was considered to indicate statistical significance.

## 3 Results

**FIGURE 1:**
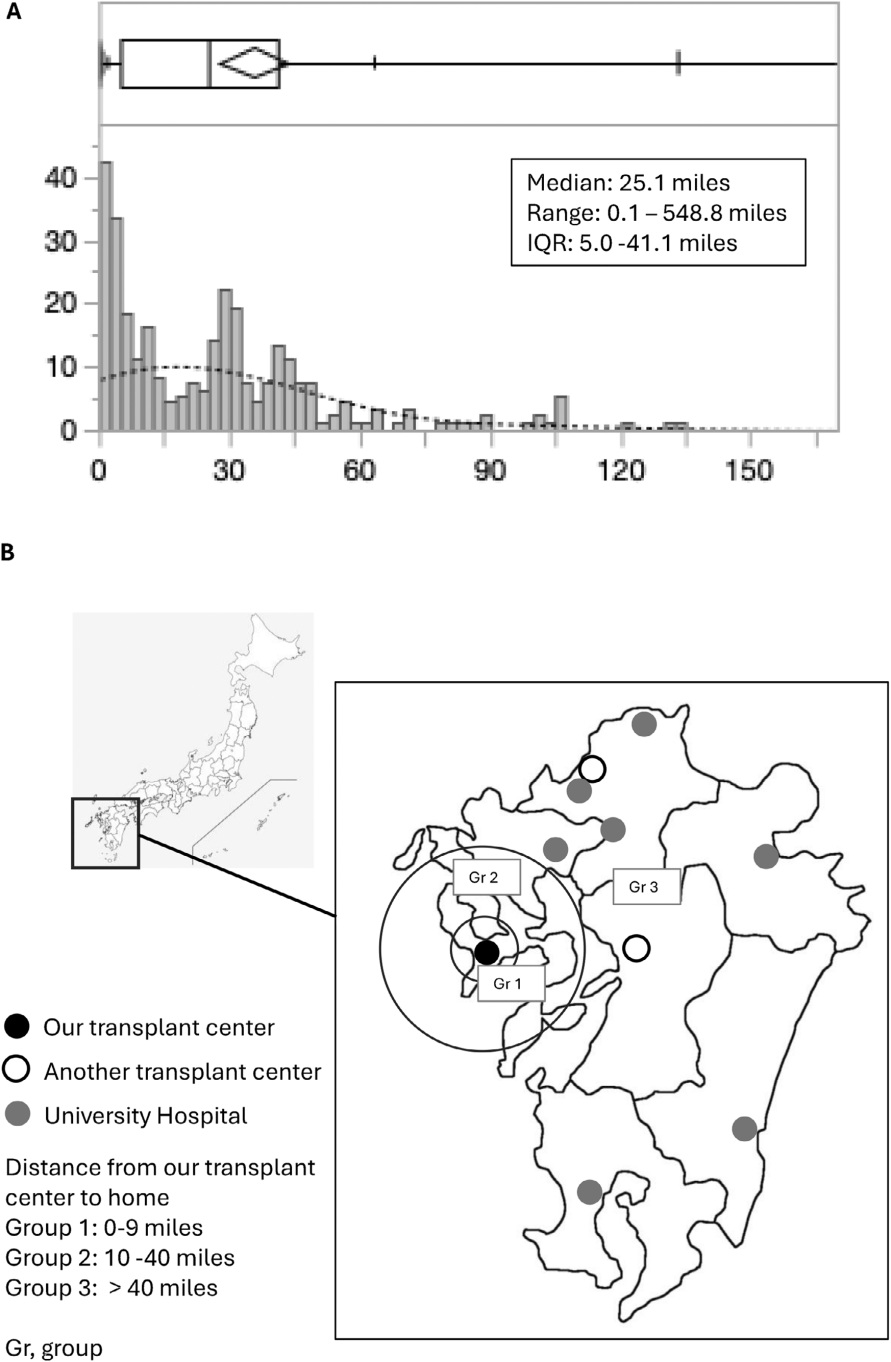
Distribution of the distances from home to our center (A) and geographical map showing the locations of transplant centers and university hospitals (B).

A total of 312 adult patients underwent LDLT at our center between January 2000 and July 2023. After the exclusion of two patients who resided outside Japan and nine who underwent retransplantation, 301 patients were finally included in the analyses. The distribution of the distances to our center is shown in Figure 1A. The median distance to the transplant center was 25.1 (5.0–41.1) miles. A geographic map showing the locations of the transplant centers and university hospitals is shown in Figure 1B. The patients were categorized as follows: Group 1, < 10 miles (*n* = 104); Group 2, 10–40 miles (*n* = 121); and Group 3, > 40 miles (*n* = 76).

### 3.1 Patient Characteristics According to Distance

No significant differences were observed between the groups in terms of recipient age and sex, MELD score, pretransplant intensive care unit (ICU) stay, portal vein thrombosis, ABO compatibility, graft type, and GRWR (Table 1). The median BMI and incidence of hepatitis B infection were significantly higher in Group 1 than in Group 2 (*p* = 0.015 and *p* = 0.022, respectively). The median donor age was significantly higher in Group 3 than in Group 2 (*p* = 0.034). With regard to operative variables, the warm ischemia time was significantly longer in Group 1 than in Groups 2 and 3 (*p* = 0.032 and *p* = 0.033, respectively). Distances did not differ significantly over the time period of the study.

**TABLE 1:** Patient characteristics according to distance.

	Gr 1 (*n* = 104)	Gr 2 (*n* = 121)	Gr 3 (*n* = 76)	Gr1 versus Gr2	Gr1 versus Gr3	Gr2 versus Gr3
				*p*	*p*	*p*
Recipient age, years	58 (52–63)	57 (50–63)	57 (50–63)	0.197	0.299	0.913
Sex, female	44 (42.3%)	49 (40.5%)	37 (48.7%)	0.783	0.396	0.259
Body mass index	24.6 (21.0–27.1)	22.5 (20.5–25.6)	23.1 (20.9–26.3)	0.015	0.408	0.212
Diagnosis						
Hepatocellular carcinoma	44 (42.3%)	45 (37.2%)	31 (40.8%)	0.434	0.838	0.613
Hepatitis B	24 (23.1%)	14 (11.6%)	9 (11.8%)	0.022	0.054	0.954
Hepatitis C	28 (26.9%)	39 (32.2%)	27 (35.5%)	0.385	0.216	0.633
PBC, PSC, AIH	12 (11.5%)	22 (18.2%)	11 (14.5%)	0.165	0.560	0.498
Alcohol‐related cirrhosis	17 (16.4%)	24 (19.8%)	12 (15.8%)	0.499	0.920	0.475
NASH	15 (14.4%)	15 (12.4%)	9 (11.8%)	0.656	0.615	0.908
MELD score	16 (13–22)	17 (13–23)	15 (12–24)	0.710	0.498	0.354
Acute on chronic liver failure	5 (4.8%)	5 (4.1%)	6 (7.9%)	1	0.531	0.342
Pretransplant medical condition, ICU	11 (10.6%)	9 (7.4%)	12 (15.8%)	0.409	0.301	0.069
Portal vein thrombosis at the time of transplant	15 (14.4%)	12 (9.9%)	9 (11.8%)	0.300	0.615	0.670
Donor age, years	39 (30–53)	38 (28–52)	42.5 (33–53.8)	0.536	0.138	0.034
ABO incompatibility	17 (16.4%)	30 (24.8%)	12 (15.8%)	0.120	0.920	0.133
Graft type (left lobe)	61 (58.7%)	72 (59.5%)	35 (46.1%)	0.897	0.094	0.065
GRWR, %	0.81 (0.63–0.93)	0.76 (0.64–0.95)	0.81 (0.63–0.95)	0.771	0.922	0.870
Operation time, hours	13.4 (12.2–15.4)	13.5 (12.1–15.1)	13.7 (12.0–15.2)	0.460	0.896	0.441
Blood loss, mL	6522 (3300–12 128)	7000 (4175–10 438)	6541 (4217–14 729)	0.631	0.212	0.367
Warm ischemia time, min	43 (38–50)	40 (35–45)	39 (34–49)	0.032	0.033	0.673
Cold ischemia time, min	103 (75–123)	95 (71–125)	102 (65–126)	0.350	0.814	0.661
Transplant era						
2000–2009	40 (38.5%)	33 (27.3%)	27 (35.5%)	0.130	0.343	0.396
2010–2019	56 (53.9%)	72 (59.5%)	38 (50.0%)			
2020–2023	8 (7.6%)	16 (13.2%)	11 (14.5%)			

Abbreviations: GRWR, graft‐to‐recipient weight ratio; MELD, model for end‐stage liver disease; NASH, nonalcoholic steatohepatitis; PBC, primary biliary cholangitis; PSC, primary sclerosing cholangitis.

### 3.2 Graft and Patient Survival According to Distance to Our Transplant Center

**FIGURE 2:**
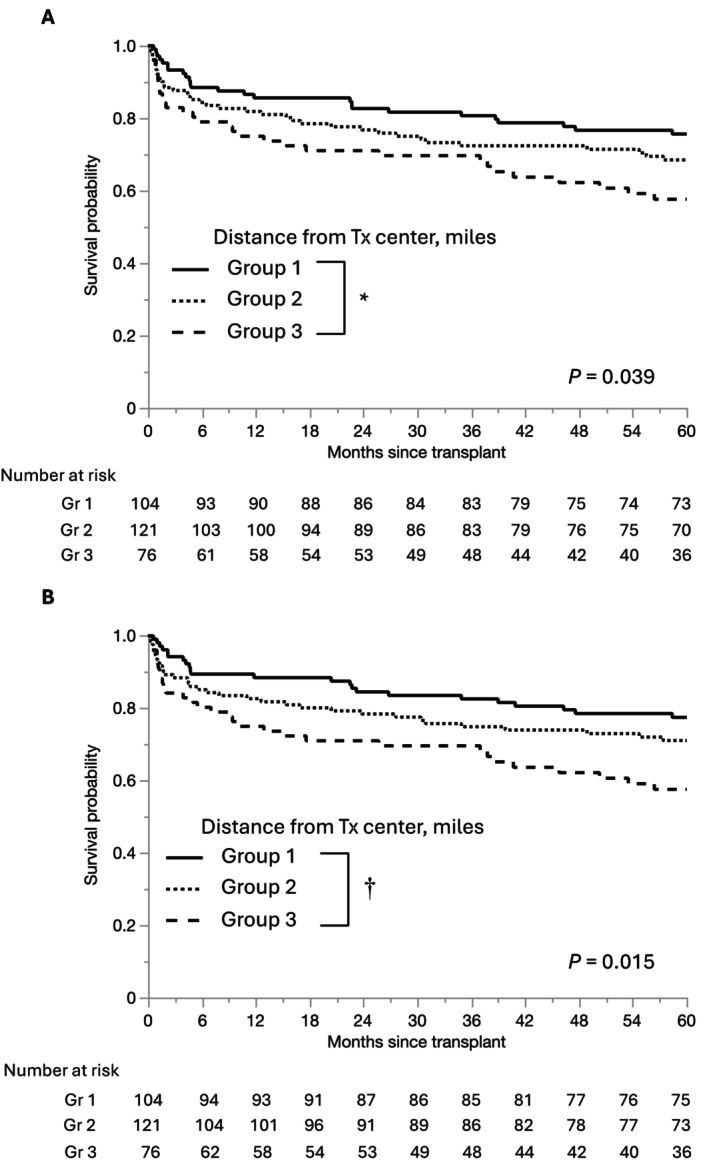
Kaplan–Meier curves of graft (A) and patient (B) survival according to distance to our transplant center. Gr, Group; Tx, transplant. *Group 1 versus Group 3; *p* = 0.010, †Group 1 versus Group 3; *p* = 0.004.

The graft survival rate was lower in Group 3 than in Group 1 (69.6% vs. 80.7% at 3 years and 57.6% vs. 75.6% at 5 years; *p* = 0.010) (Figure 2A). Likewise, the patient survival rate was significantly lower in Group 3 than in Group 1 (69.6% vs. 82.6% at 3 years and 57.6% vs. 77.5% at 5 years; *p* = 0.004) (Figure 2B).

### 3.3 Prognostic Significance of Distance to Our Transplant Center

A multivariate Cox proportional hazard model was used to identify factors associated with survival (Table 2). In the univariate analysis, infection with hepatitis C (hazard ratio [HR]: 1.578, *p* = 0.035), ACLF (HR: 2.165, *p* = 0.037), a distance to our transplant center of > 40 miles (Group 3) (HR: 2.158, *p* = 0.005), donor age (HR: 1.454, per 10 year increase, *p* < 0.001), amount of intraoperative blood loss (HR: 1.032, per 1000 mL increase, *p* < 0.001), and cold ischemia time (HR: 1.043, per 10 min increase, *p* = 0.039) were significantly associated with patient survival. In the multivariate analysis, infection with hepatitis C (HR: 1.570, *p* = 0.049), a distance to our transplant center of > 40 miles (Group 3) (HR: 1.901, *p* = 0.025), donor age (HR: 1.403, per 10 year increase, *p* < 0.001), and amount of intraoperative blood loss (HR: 1.031, per 1000 mL increase, *p* = 0.002) were independently associated with patient survival. To further confirm the prognostic significance of a distance of > 40 miles to our transplant center, PSM was conducted to compare the survival outcomes of patients in Group 3 with those in Groups 1 and 2. Although significant differences in donor age and graft type were observed between Groups 1 + 2 and Group 3 before matching, all factors were well balanced after matching (Table S1). Although significant differences in graft survival were not observed after PSM (*p* = 0.148), patient survival was significantly worse in Group 3 than in Groups 1 + 2 (*p* = 0.029) (Figure S1).

**TABLE 2:** Cox hazard model for patient survival.

	HR	*p*	HR	*p*
Recipient age, per 10 years increase	1.209 (0.986–1.512)	0.082		
Sex, female	0.742 (0.481–1.146)	0.179		
Body mass index, per 10 kg/m^2^ increase	1.011 (0.731–1.451)	0.951		
Diagnosis				
Hepatocellular carcinoma	1.070 (0.701–1.633)	0.755		
Hepatitis B	0.552 (0.277–1.101)	0.092		
Hepatitis C	1.578 (1.031–2.414)	0.035	1.570 (01.003–2.459)	0.049
PBC, PSC, AIH	0.985 (0.547–1.775)	0.959		
Alcohol‐related cirrhosis	0.853 (0.482–1.510)	0.585		
NASH	0.796 (0.399–1.586)	0.516		
MELD score, per 10 increase	1.018 (0.992–1.044)	0.169		
Acute on chronic liver failure	2.165 (1.046–4.482)	0.037	1.628 (0.729–3.632)	0.234
Pretransplant medical condition, ICU	1.347 (0.716–2.535)	0.355		
Portal vein thrombosis at the time of transplant	0.994 (0.515–1.921)	0.987		
Distance from Tx center				
Gr 1	Ref	—	Ref	—
Gr 2	1.383 (0.815–2.348)	0.230	1.444 (0.820–2.544)	0.204
Gr 3	2.158 (1.258–3.703)	0.005	1.901 (1.083–3.335)	0.025
Donor age, per 10 years increase	1.454 (1.238–1.715)	< 0.001	1.403 (1.179–1.674)	< 0.001
ABO incompatibility	1.022 (0.602–1.734)	0.937		
Graft type (left lobe)	0.937 (0.616–1.425)	0.761		
GRWR, per 1.0% increase	0.523 (0.200–1.290)	0.173		
Operation time, per 1 h increase	1.017 (0.949–1.081)	0.608		
Blood loss, per 1000 mL increase	1.032 (1.012–1.048)	< 0.001	1.031 (1.009–1.049)	0.002
Transfusion				
Red blood cells, per 10 U increase	1.119 (1.062–1.169)	< 0.001		
Fresh‐frozen plasma, per 10 U increase	1.029 (0.930–1.125)	0.557		
Platelets, U, per 10 U increase	1.112 (0.970–1.263)	0.126		
Warm ischemia time, per 10 min increase	1.137 (0.920–1.379)	0.215		
Cold ischemia time, per 10 min increase	1.043 (1.000–1.083)	0.039	1.026 (0.983–1.068)	0.212
Transplant era		0.604		
2000–2009	Ref	—		
2010–2019	1.007 (0.645–1.574)	0.974		
2020–2023	0.656 (0.272–1.578)	0.346		

Abbreviations: GRWR, graft‐to‐recipient weight ratio; MELD, model for end‐stage liver disease; NASH, nonalcoholic steatohepatitis; PBC, primary biliary cholangitis; PSC, primary sclerosing cholangitis.

### 3.4 Short‐ and Long‐Term Survival According to Distance to Our Transplant Center

**FIGURE 3:**
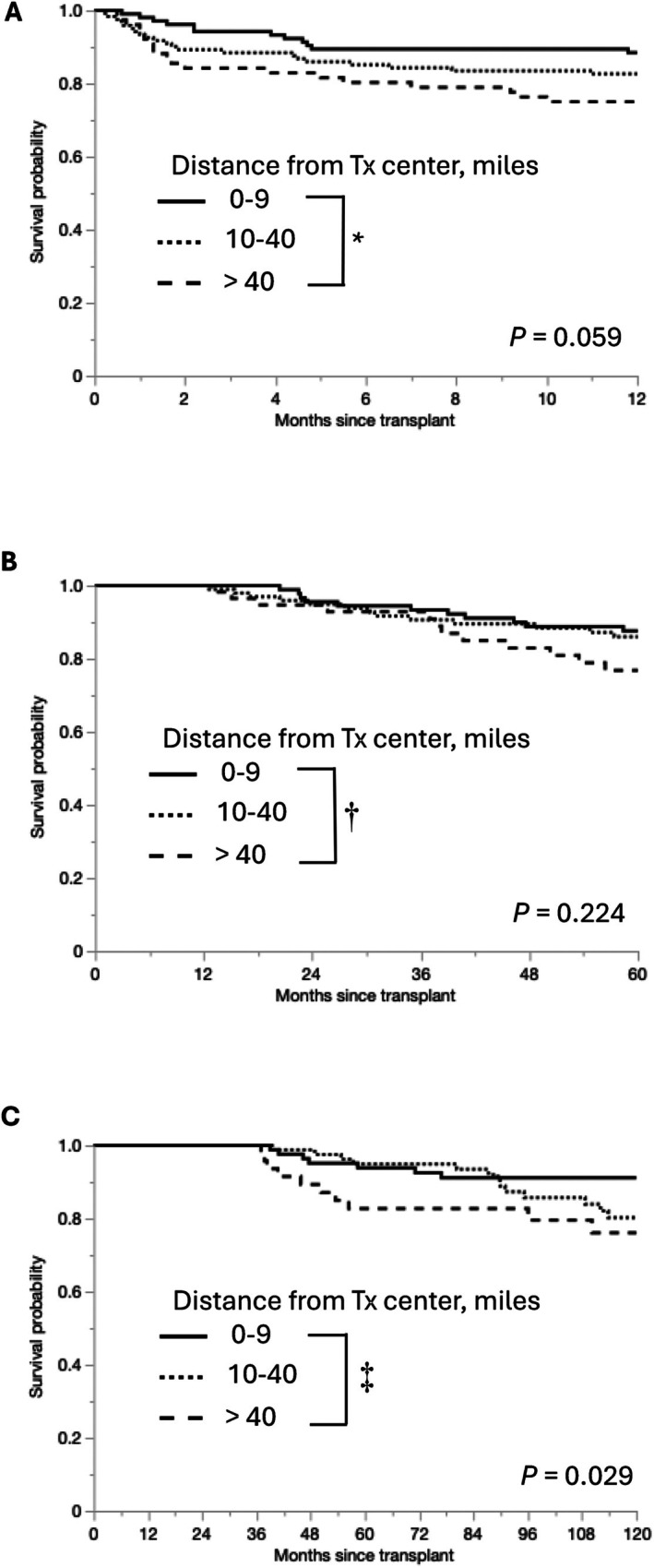
Short‐ and long‐term survival according to distance to our transplant center. Kaplan–Meier curves of 1‐year patient survival (A), 1‐year conditional survival (B), and 3‐year conditional survival (C) according to distance to our transplant center. Tx, transplant. *Group 1 versus Group 3; *p* = 0.016, †Group 1 versus Group 3; *p* = 0.099, ‡Group 1 versus Group 3; *p* = 0.027.

To analyze the impact of distance on short‐term survival outcomes, 1‐year survival rates were compared between the groups. The 1‐year patient survival rate was significantly lower in Group 3 than in Group 1 (75.0% vs. 88.5%, *p* = 0.016) (Figure 3A). Conditional survival analyses were also performed. Although the 1‐year conditional survival rate did not differ significantly between Groups 1 and 3 (*p* = 0.099) (Figure 3B), the three‐year conditional survival rate was significantly lower in Group 3 than in Group 1 (*p* = 0.027) (Figure 3C). Likewise, although the differences were not statistically significant, the analyses of 1‐year patient survival and 3‐year conditional survival in the matched cohort indicated worse short‐ and long‐term survival outcomes as well (Figure S2).

### 3.5 Surgical Complications and Locations of Patient Deaths

Because a lower 1‐year survival rate was observed in Group 3 than in Group 1, the incidence of surgical complications was evaluated (Table 3). The incidence of hepatic artery thrombosis was significantly higher in Group 3 than in Groups 1 and 2, which might have contributed to the inferior 1‐year survival rate observed in this group. In the matched cohort, significant differences in the incidence rates of surgical complications were not found (Table S2). There were no significant intergroup differences in other surgical complications. The rate of in‐hospital mortality was higher in Group 3 (19.7%) than in Groups 1 (8.7%) and 2 (13.2%), but this difference did not reach statistical significance (*p* = 0.099) (Table 3). There were no significant differences in liver function including AST, ALT, bilirubin, and INR at the time of discharge between the groups (Table S3). The proportion of patients who were transferred to another hospital after transplantation was higher in Group 3 compared with other groups (Group 1: 19.8%, Group 2: 28.0%, and Group 3: 41.5%, *p* = 0.025) (Table S3).

**TABLE 3:** Posttransplant complications and location of death.

	Gr 1 (*n* = 104)	Gr 2 (*n* = 121)	Gr 3 (*n* = 76)	*p*
Hepatic artery thrombosis, *n* (%)	1 (1%)	3 (2.5%)	7 (9.2%)	0.010
Portal vein thrombosis, *n* (%)	19 (18.3%)	12 (9.9%)	10 (13.2%)	0.189
Biliary complications, *n* (%)	21 (20.2%)	23 (19.0%)	10 (13.2%)	0.442
Acute cellular rejection, *n* (%)	34 (32.7%)	38 (31.4%)	23 (30.3%)	0.941
Early allograft dysfunction, *n* (%)	31 (29.8%)	44 (36.7%)	28 (36.8%)	0.502
Small‐for‐size syndrome, *n* (%)	31 (29.8%)	29 (24.0%)	21 (27.6%)	0.608
90‐day mortality, *n* (%)	6 (5.8%)	14 (11.6%)	12 (15.8%)	0.089
In‐hospital mortality	9 (8.7%)	16 (13.2%)	15 (19.7%)	0.099
Number of patients who died after discharge	22	28	20	
Location of death				
Our transplant center	16 (72.7%)	9 (32.1%)	6 (30.0%)	0.005
Other hospital	6 (27.3%)	19 (67.9%)	14 (70.0%)	

We also investigated the location of death in patients who were discharged after LDLT. The proportion of patients who died at our center significantly decreased as the distance to our center increased (Group 1: 72.7%, Group 2: 32.1%, Group 3: 30.0%; *p* = 0.005) (Table 3).

### 3.6 Causes of Death, Retransplant Rates, and Long‐Term Follow‐Up Status

The causes of death or graft failure according to distance from the transplant center at various time points are shown in Table 4. No significant differences in the causes of death or graft failure were observed among the three groups. No patients in Group 3 underwent retransplantation 1 year after LDLT. In the analyses of the matched cohort, the causes of death or graft failure were similar in Group 1 + 2 and Group 3 (Table S4). The retransplant rate was significantly higher in Group 1 + 2 (10.8%) than in Group 3 (1.4%) (*p* = 0.034). The actual number of follow‐up visits per year 3 years after transplantation was fewer in Group 3 compared with that in the other groups (*p* < 0.001) (Table S4).

**TABLE 4:** Causes and locations of death at different time points.

	Within 1 year				1–3 years				After 3 years			
	Gr 1	Gr 2	Gr 3	*p*	Gr 1	Gr 2	Gr 3	*p*	Gr 1	Gr 2	Gr 3	*p*
	(*n* = 104)	(*n* = 121)	(*n* = 76)		(*n* = 89)	(*n* = 99)	(*n* = 57)		(*n* = 82)	(*n* = 82)	(*n* = 47)	
Causes of death or graft failure												
Primary graft failure	0 (0%)	3 (2.5%)	3 (4.0%)	0.154	0 (0%)	1 (1.0%)	0 (0%)	0.477	1 (1.2%)	2 (2.4%)	1 (2.1%)	0.841
Infectious disease	8 (7.7%)	12 (9.9%)	8 (10.5%)	0.775	1 (1.1%)	0 (0%)	2 (3.5%)	0.158	1 (1.2%)	4 (4.9%)	1 (2.1%)	0.350
PTLD or malignancy	1 (1.0%)	0 (0%)	1 (1.3%)	0.488	2 (2.3%)	5 (5.1%)	0 (0%)	0.173	6 (7.3%)	5 (6.1%)	4 (8.5%)	0.873
Recurrence of primary disease	0 (0%)	0 (0%)	1 (1.3%)	0.227	0 (0%)	2 (2.0%)	2 (3.5%)	0.244	0 (0%)	3 (3.7%)	1 (2.1%)	0.227
Vascular complications	3 (2.9%)	1 (0.8%)	2 (2.6%)	0.491	0 (0%)	1 (1.0%)	0 (0%)	0.477	0 (0%)	0 (0%)	1 (2.1%)	0.173
Rejection	1 (1.0%)	1 (0.8%)	2 (2.6%)	0.516	1 (1.1%)	0 (0%)	0 (0%)	0.415	1 (1.2%)	0 (0%)	0 (0%)	0.454
Retransplant rate	3 (2.9%)	1 (0.8%)	1 (1.3%)	0.467	1 (1.1%)	3 (3.0%)	0 (0%)	0.318	0 (0%)	3 (3.7%)	0 (0%)	0.091

Abbreviation: PTLD, posttransplant lymphoproliferative disorders.

### 3.7 Association Between Survival and Distance to Specialized Medical Service

**FIGURE 4:**
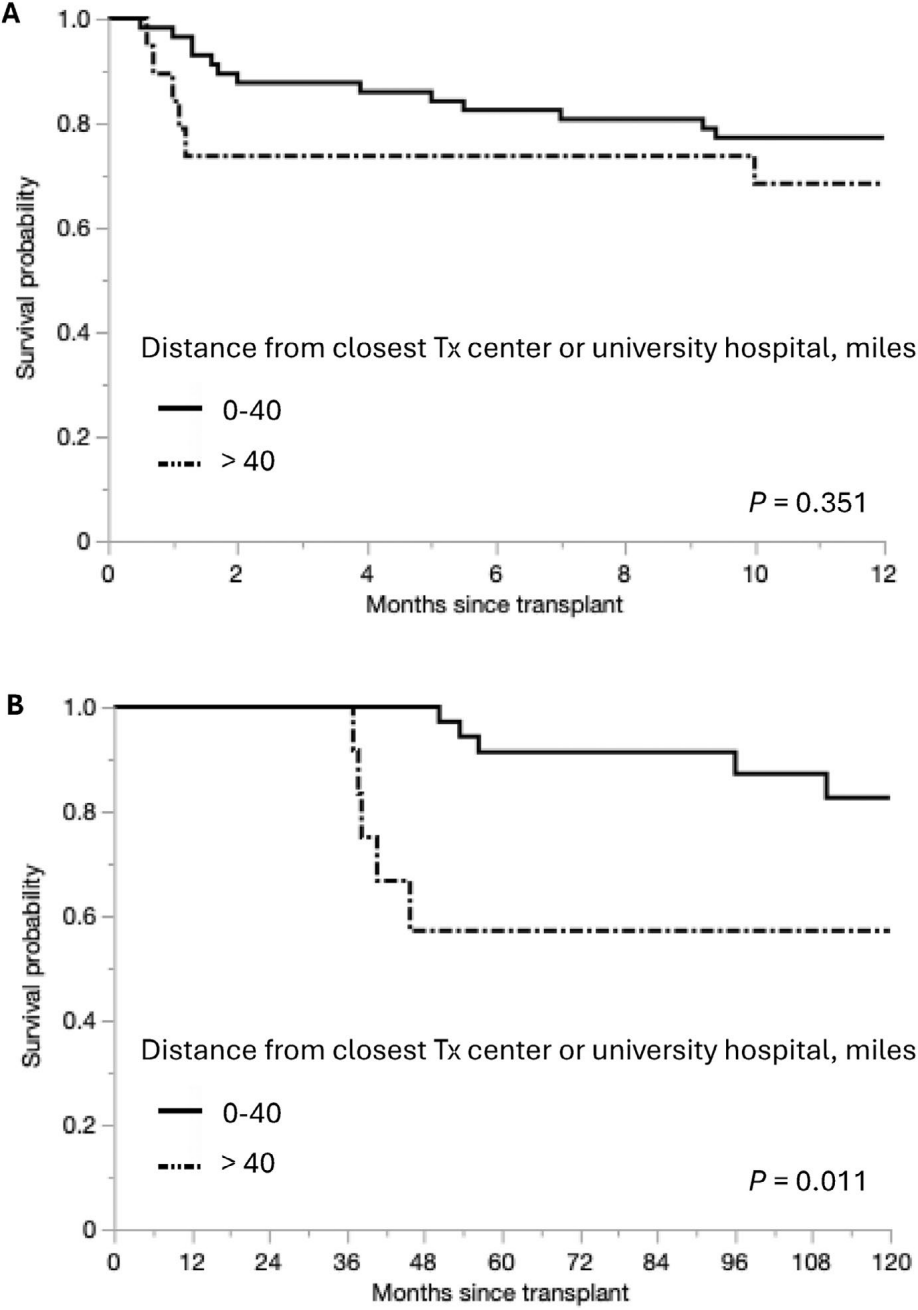
Short‐ and long‐term survival of patients in Group 3 according to distance from home to the nearest transplant center or university hospital. Kaplan–Meier curves of 1‐year patient survival (A) and 3‐year conditional survival (B) according to distance to the nearest specialized medical center. Tx, transplant.

The 76 patients in Group 3 were further divided into two groups according to their distance to the nearest transplant center or university hospital; 57 patients resided < 40 miles from the nearest specialized medical service and the remaining 19 resided > 40 miles from the nearest specialized medical service. Although there were no significant differences in the 1‐year patient survival rates between the two groups (Figure 4A), 3‐year conditional survival analysis showed the inferior survival of patients residing > 40 miles from the nearest specialized medical service (Figure 4B).

## 4 Discussion

In Japan, LDLT is commonly performed for the treatment of patients with end‐stage liver disease because of the severe shortage of liver grafts from deceased donors [20, 21]. Because of the surgical expertise and specialized medical care required, LDLT is performed at only 23 certified facilities in Japan. Therefore, some patients have to travel long distances to undergo LDLT and receive posttransplant care. As treatment delays due to limited access to specialized medical care can be fatal in transplant recipients, we hypothesized that the distance from home to the transplant center might be associated with survival following LDLT. To the best of our knowledge, this is the first study to focus on the influence of distance from home to the transplant center on survival following LDLT. Our results indicated that a greater distance to our center was associated with worse survival, and this finding was validated using a multivariate Cox regression model and PSM. Conditional survival analysis showed that a greater distance to our center negatively affected both short‐ and long‐term survival. Patients residing at a greater distance from our center had a lower chance of retransplantation compared to those residing closer to our center.

Previously, Firozvi et al. reported similar one‐year posttransplant survival rates for 15 patients living > 3 h drive away and 51 patients living < 3 h drive away from the transplant center [11]. However, the number of patients included in the study was limited and long‐term outcomes were not assessed. A recent study from Korea examined the influence of medical accessibility on long‐term outcomes of LT and found no differences in posttransplant survival according to distance to the transplant center [12]. This study excluded cases of early posttransplant deaths, prolonged hospitalizations, and those from remote islands, which may explain why the findings differ from the results of our study. The present study showed significant differences in 1‐year patient survival rates between Groups 1 and 3, and a higher rate of in‐hospital mortality in Group 3. One possible reason for this is that the incidence of hepatic artery thrombosis, which may compromise graft outcomes, was higher in Group 3 by chance. To minimize the influence of early surgical complications such as hepatic artery thrombosis and patient heterogeneity on long‐term survival outcomes, conditional survival analysis was performed, which showed that 3‐year patient survival in Group 3 was inferior to that in Group 1. The functional recovery including stable liver function and exercise capacity at the time of discharge is essential for ensuring long‐term survival outcomes. Because we recommended that the patients who lived far from our center be transferred to a nearby hospital before being discharged to their home regardless of their liver function, the proportion of patients who were transferred to another hospital was the highest in Group 3. Considering that there were no significant differences in liver function at the time of discharge between the groups, it is possible that the postdischarge course might have an even greater influence on long‐term outcomes.

Since 3‐year conditional survival analysis found significant differences in long‐term survival outcomes according to the distance, we further investigated the subsequent follow‐up visits to our center among patients who had survived over 3 years after LDLT. As shown in Table S4, the number of follow‐up visits in Group 3 was fewer than that in the other groups. The less frequent follow‐up potentially resulted in worse long‐term outcomes in patients living far from our center. As shown in Figure 4, PTLD and other malignancies were the leading causes of death after 3 years since LDLT in Group 3. Of 4 patients who died from malignancies after 3 years since LDLT, 2 patients were diagnosed with PTLD at a nearby hospital and were initiated treatment at the same facility. Although the clinical course leading to the diagnosis and the specific details of treatment were unclear, those cases underscore the importance of meticulous surveillance at transplant centers to facilitate early detection and timely initiation of appropriate treatment. Furthermore, seven patients residing > 40 miles away no longer visited our center 3 years after transplantation. Of those, 3 patients died from uncertain causes in another hospital. On the other hand, the other 4 patients are still alive undergoing regular follow‐up at another university hospital. These findings highlight the importance of seamless follow‐up by specialized medical services to optimize long‐term outcomes after LDLT regardless of the residential area of recipients.

The subgroup analysis of Group 3 showed that patients living > 40 miles from the nearest specialized medical center experienced worse long‐term survival outcomes. Transplant centers and university hospitals may provide specialized care to LT recipients who live far from their original transplant center. While we did not have precise data on the actual number of visits to other transplant centers or university hospitals by patients in our cohort, our findings suggest that patients living relatively close to other transplant centers or university hospitals have favorable survival outcomes, even if they are located far from the primary transplant center.

This study has some limitations. First, this was a retrospective study based on a limited number of patients from a single center. Therefore, further validation is necessary to determine whether the results of this study can be generalized to cohorts from other transplant centers. Although previous studies using national databases, such as the UNOS database and UK transplant registry, included larger patient populations, zip codes were used to determine distance [2, 3, 5], whereas the present study used the specific home addresses of the patients, allowing for a more accurate assessment of distance. Second, owing to the wide variety of transport used in Japan, it is difficult to determine actual travel times. Therefore, we categorized and compared groups based on the straightline distance from the home of the patient to the transplant center.

In conclusion, this is the first study to investigate the influence of distance to the transplant center on survival following LDLT in Japan. A greater distance to the transplant center was associated with worse survival outcomes after LDLT. For LDLT recipients residing far from the primary transplant center, while routine follow‐up care in collaboration with local hospitals might be generally sufficient, the event of any abnormalities necessitates prompt and comprehensive evaluation at the transplant center or specialized medical center. Further nationwide investigations are needed to validate our findings and determine the geographic areas in Japan with limited access to specialized services and posttransplant care, resulting in compromised survival outcomes.

## Author Contributions

**Hajime Matsushima:** conceptualization, methodology, investigation, writing – original draft, writing – review and editing, formal analysis, visualization. **Akihiko Soyama:** writing – review and editing. **Takanobu Hara:** investigation, data curation. **Ayaka Kinoshita:** data curation. **Takashi Hamada:** data curation. **Kazushige Migita:** data curation. **Ayaka Satoh:** data curation. **Hajime Imamura:** data curation. **Tomohiko Adachi:** data curation. **Susumu Eguchi:** supervision.

## Ethics Statement

This study was approved by the Ethics Committee of Nagasaki University Hospital (Approval No. 20012022‐2) and conducted in accordance with the principles outlined in the Declaration of Helsinki. The requirement for informed consent was waived owing to the retrospective nature of the study.

## Conflicts of Interest

S.E. is an editorial board member of Annals of Gastroenterological Surgery.

## Supporting information


**Figure S1.** Graft (A) and patient (B) survival after propensity score matching.
**Figure S2**. One‐year survival and conditional survival analyses in the matched cohort.
**Figure S3**. Percentages of patients who were transferred to another hospital after LDLT.
**Figure S4**. Number of visits to our transplant center per a year after 3 years since LDLT.

**Table S1.** Patient characteristics before and after propensity score matching.

**Table S2.** Posttransplant complications in the matched cohort.

**Table S3.** Graft function at the time of discharge.

**Table S4.** Causes of death or graft failure and retransplant rate in the matched cohort.

## Data Availability

The requirement for informed consent was waived owing to the retrospective nature of the study.
