# Supplementary material for: Association Between Distance to the Transplant Center and Survival Following Living Donor Liver Transplantation
Source: Ann Gastroenterol Surg. 2025 Jun 9;9(6):1322–33. doi: 10.1002/ags3.70051 (PMC12586949; doi:10.1002/ags3.70051)
Supplement: Supplementary file 1 — Figure S1. Graft (A) and patient (B) survival after propensity score matching. Figure S2. One‐year survival and conditional survival analyses in the matched cohort. Figure S3. Percentages of patients who were transferred to another hospital after LDLT. Figure S4. Number of visits to our transplant center per a year after 3 years since LDLT. [file AGS3-9-1322-s002.pptx]

## Slide 1
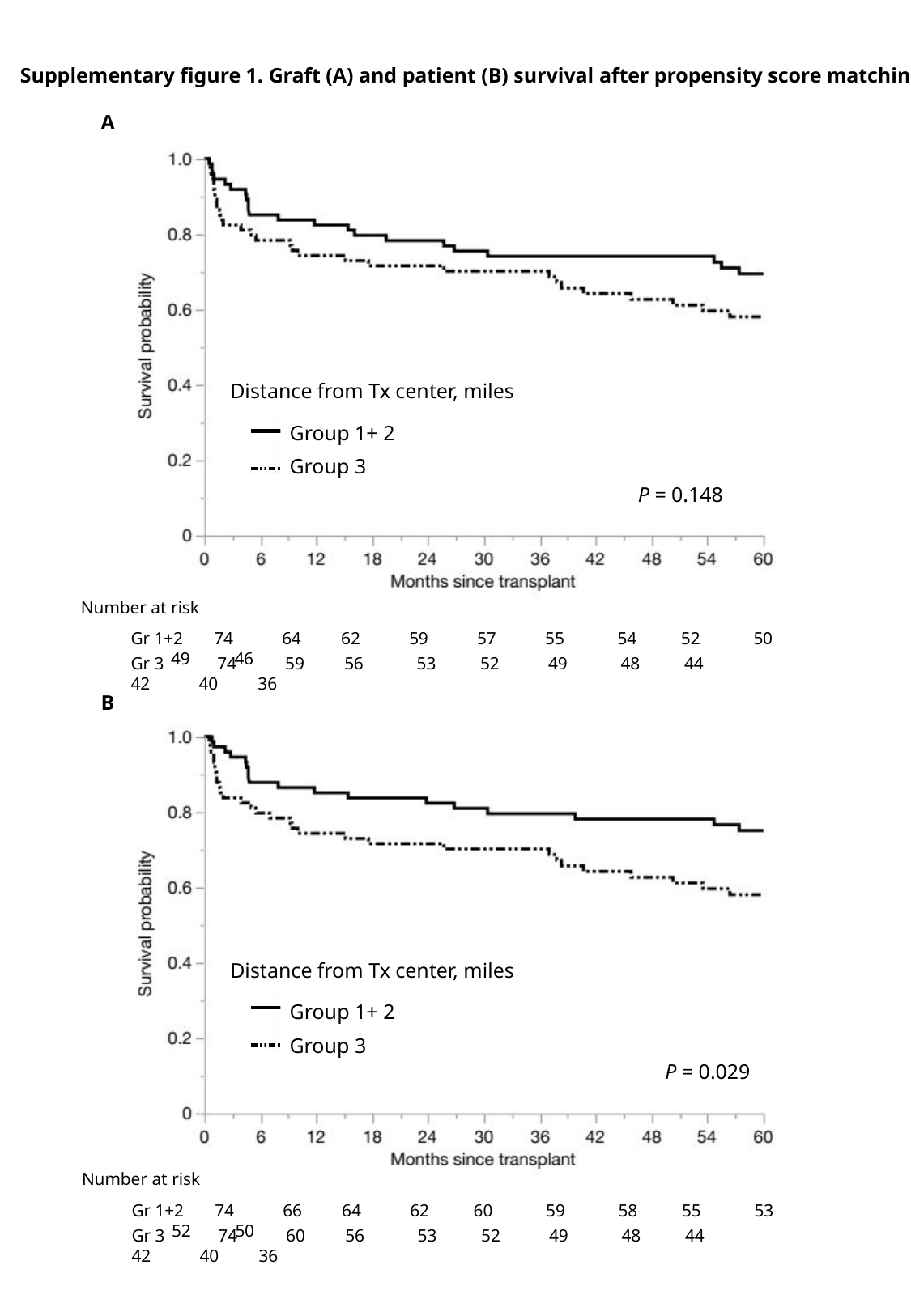

Supplementary figure 1. Graft (A) and patient (B) survival after propensity score matching
A
Distance from Tx center, miles
Group 1+ 2
Group 3
P = 0.148
Number at risk
Gr 1+2 74 64 62 59 57 55 54 52 50 49 46
Gr 3 74 59 56 53 52 49 48 44 42 40 36
B
Distance from Tx center, miles
Group 1+ 2
Group 3
P = 0.029
Number at risk
Gr 1+2 74 66 64 62 60 59 58 55 53 52 50
Gr 3 74 60 56 53 52 49 48 44 42 40 36

## Slide 2
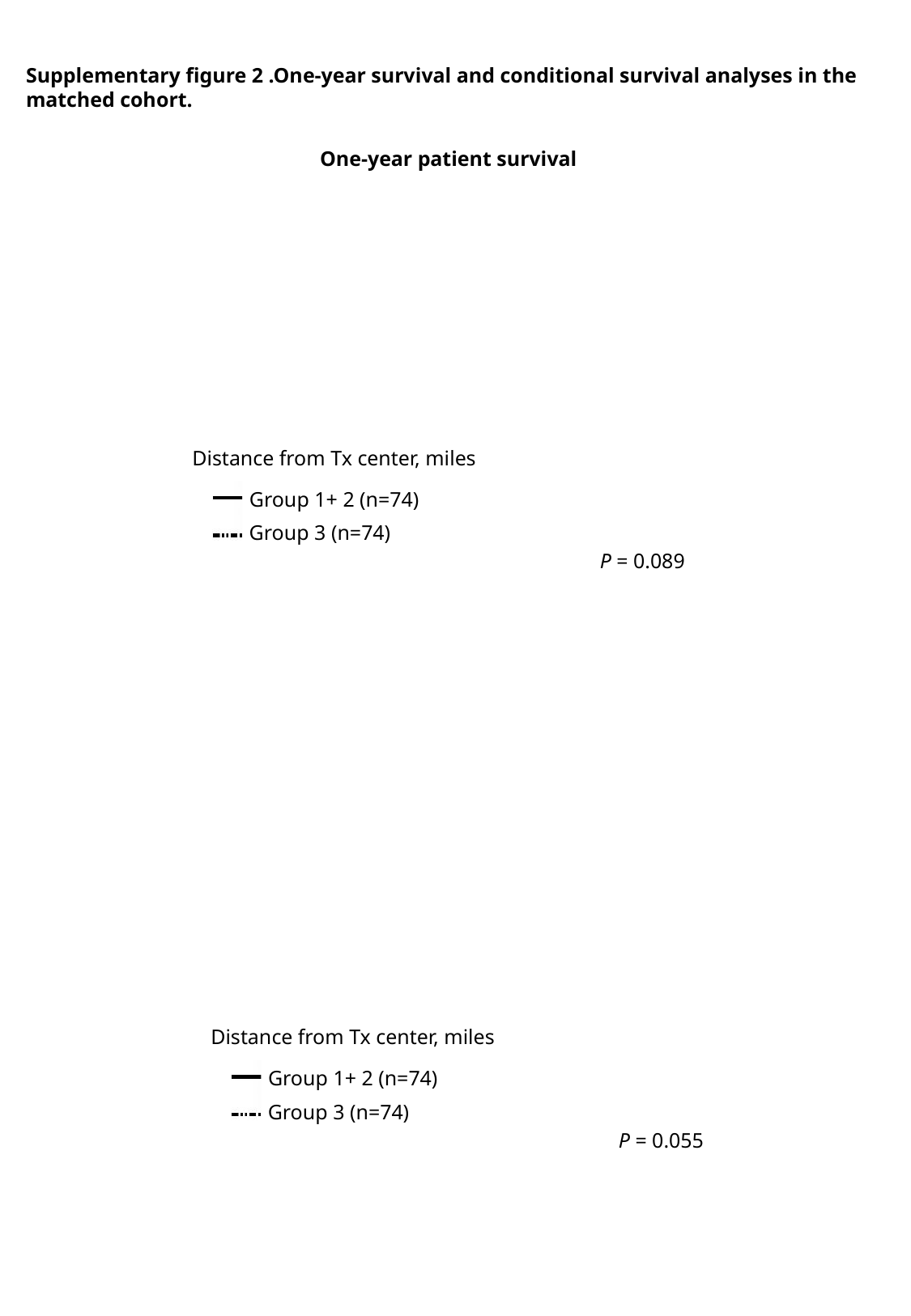

Supplementary figure 2 .One-year survival and conditional survival analyses in the
matched cohort.
One-year patient survival
Distance from Tx center, miles
Group 1+ 2 (n=74)
Group 3 (n=74)
P = 0.089
Distance from Tx center, miles
Group 1+ 2 (n=74)
Group 3 (n=74)
P = 0.055

## Slide 3
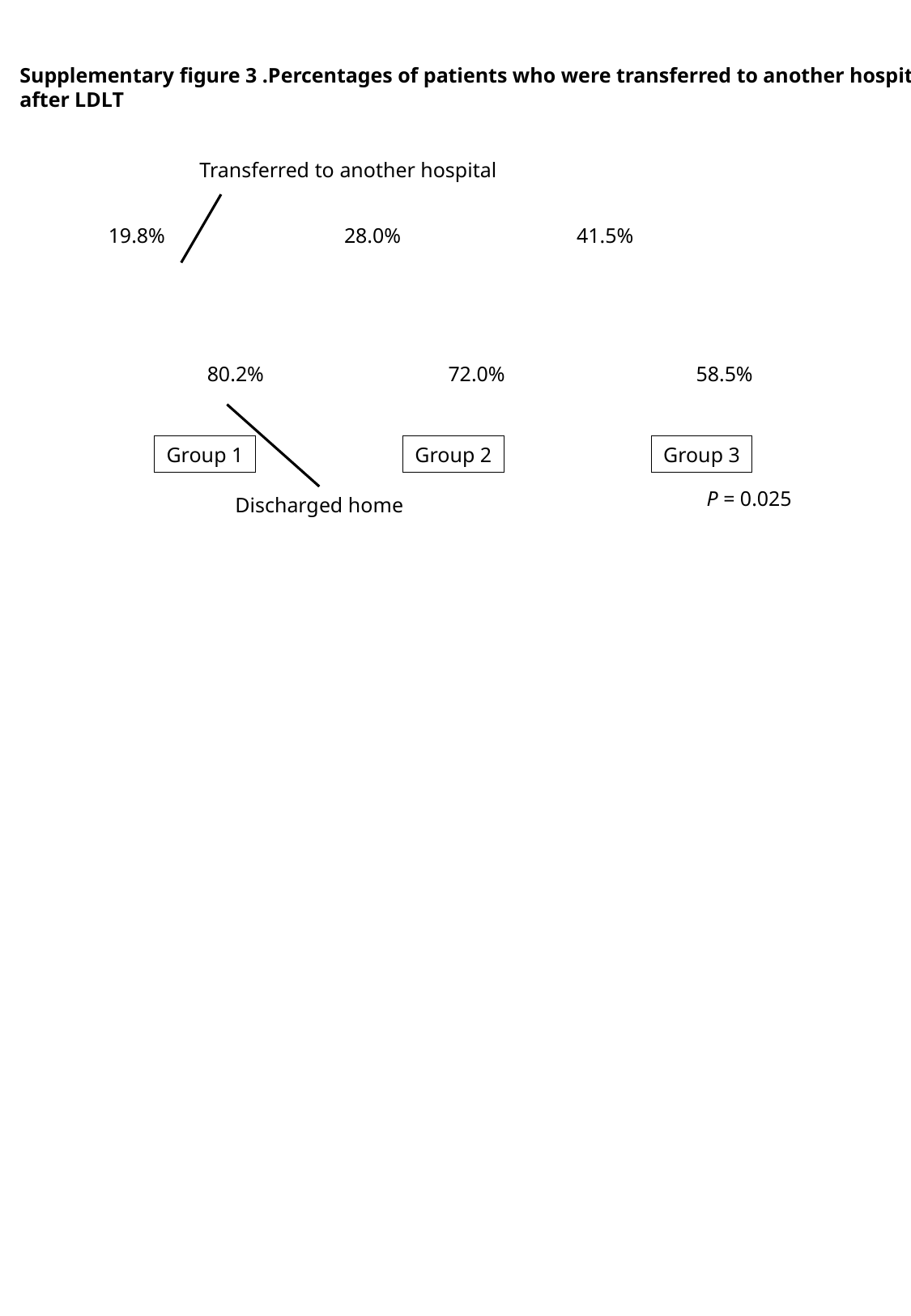

Supplementary figure 3 .Percentages of patients who were transferred to another hospital
after LDLT
Transferred to another hospital
19.8%
28.0%
41.5%
80.2%
58.5%
72.0%
Group 1
Group 2
Group 3
P = 0.025
Discharged home

## Slide 4
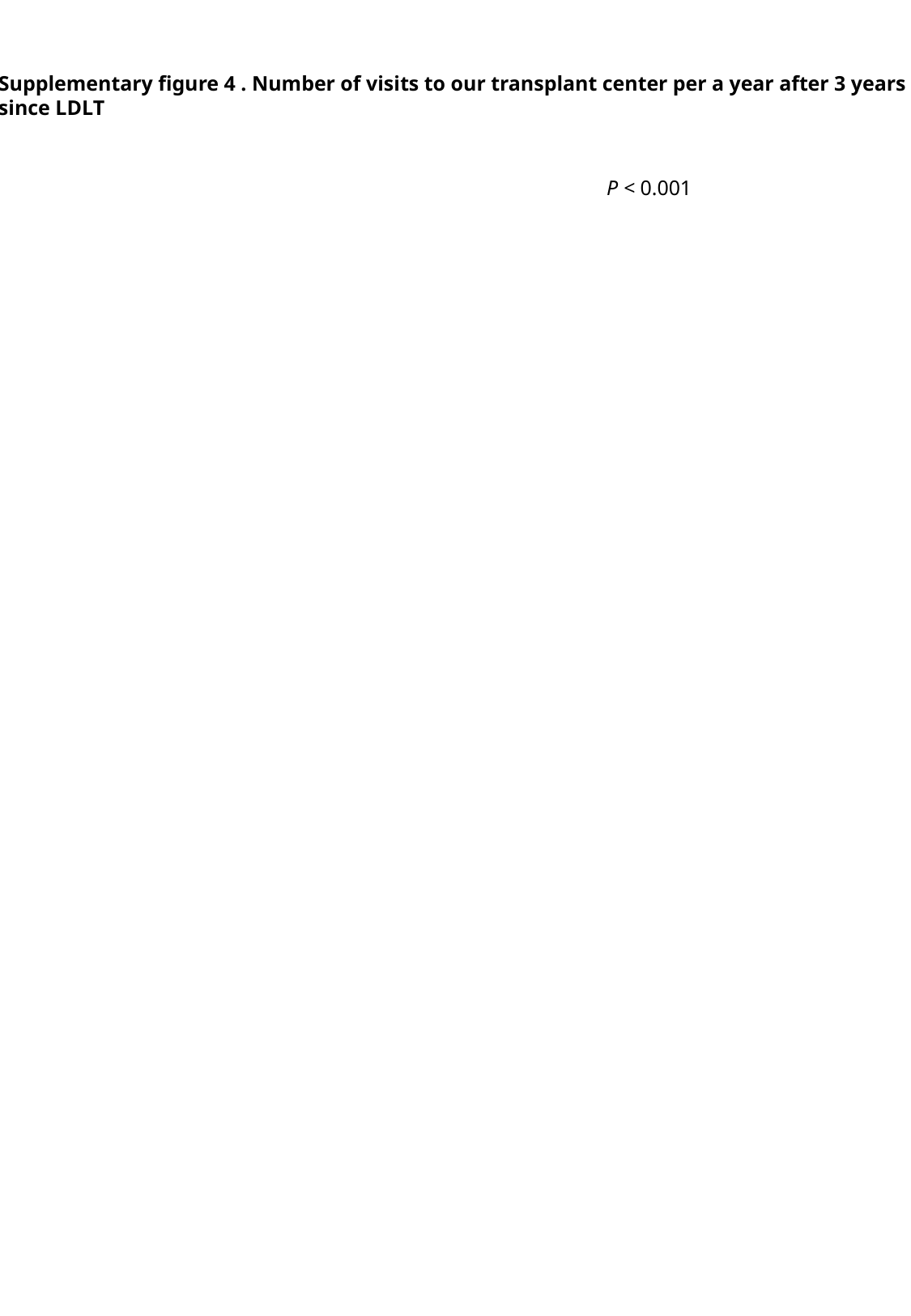

Supplementary figure 4 . Number of visits to our transplant center per a year after 3 years
since LDLT
P < 0.001
