# Supplementary material for: Association Between Distance to the Transplant Center and Survival Following Living Donor Liver Transplantation
Source: Ann Gastroenterol Surg. 2025 Jun 9;9(6):1322–33. doi: 10.1002/ags3.70051 (PMC12586949; doi:10.1002/ags3.70051)
Supplement: Supplementary file 2 — Table S1. Patient characteristics before and after propensity score matching. [file AGS3-9-1322-s003.docx]

| **Supplementary Table1. Patient characteristics before and after propensity score matching** | | | | | | |  |
| --- | --- | --- | --- | --- | --- | --- | --- |
|  | **Before matching** | | |  | **After matching** | | |
|  | **Gr 1+ Gr 2(n=225)** | **Gr 3 (n=76)** | **p-value** |  | **Gr 1+ Gr 2(n=74)** | **Gr 3 (n=74)** | **p-value** |
| Recipient age, y | 57 (51-63) | 57 (50-63) | 0.631 |  | 55 (50-62.3) | 57 (50-63.3) | 0.606 |
| Sex, female | 93 (41.3%) | 37 (48.7%) | 0.263 |  | 30 (40.5%) | 37 (50.0%) | 0.248 |
| Body mass index | 23.4 (20.7-26.2) | 23.1 (20.9-26.3) | 0.759 |  | 23.5 (21.0-25.8) | 23.3 (21.1-26.4) | 0.818 |
| Diagnosis |  |  |  |  |  |  |  |
| Hepatocellular carcinoma | 89 (39.6%) | 31 (40.8%) | 0.849 |  | 34 (46.0%) | 30 (40.5%) | 0.507 |
| Hepatitis B | 38 (16.9%) | 9 (11.8%) | 0.295 |  | 13 (17.6%) | 9 (12.2%) | 0.355 |
| Hepatitis C | 67 (29.8%) | 27 (35.5%) | 0.350 |  | 29 (39.2%) | 26 (35.1%) | 0.610 |
| PBC,PSC,AIH | 34 (15.1%) | 11 (14.5%) | 0.893 |  | 7 (9.5%) | 11 (14.9%) | 0.314 |
| Alcohol-related cirrhosis | 41 (18.2%) | 12 (15.8%) | 0.630 |  | 9 (12.2%) | 12 (16.2%) | 0.480 |
| NASH | 30 (13.3%) | 9 (11.8%) | 0.738 |  | 8 (10.8%) | 9 (12.2%) | 0.797 |
| MELD score | 17 (13-22.5) | 15 (12-24) | 0.364 |  | 16 (12-21) | 15 (12-24) | 0.819 |
| Acute on chronic liver failure | 10 (4.4%) | 6 (7.9%) | 0.247 |  | 4 (5.4%) | 5 (6.8%) | 1 |
| Pretransplant medical condition, ICU | 20 (8.9%) | 12 (15.8%) | 0.092 |  | 9 (12.2%) | 11 (14.9%) | 0.631 |
| Portal vein thrombosis at the time of transplant | 27 (12.0%) | 9 (11.8%) | 0.583 |  | 8 (10.8%) | 9 (12.2%) | 0.797 |
| Donor age, y | 38 (29-52.5) | 42.5 (33-53.8) | 0.041 |  | 43 (32-54.3) | 42.5 (32.8-54.5) | 0.863 |
| ABO incompatibility | 47 (20.9%) | 12 (15.8%) | 0.333 |  | 10 (13.5%) | 12 (16.2%) | 0.644 |
| Graft type (left lobe) | 133 (59.1%) | 35 (46.1%) | 0.048 |  | 36 (48.7%) | 34 (46.0%) | 0.742 |
| GRWR, % | 0.772 (0.637-0.944) | 0.806 (0.628-0.953) | 0.881 |  | 0.787 (0.664-0.952) | 0.806 (0.625-0.966) | 0.790 |
| Operation time, hr | 13.4 (12.1-15.3) | 13.7 (12.0-15.2) | 0.598 |  | 13.6 (12.2-15.5) | 13.7 (12.0-15.4) | 0.980 |
| Blood loss, mL | 6694 (3800-11305) | 6541 (4217-14729) | 0.233 |  | 5235 (3444-12042) | 6470 (4184-13967) | 0.120 |
| Warm ischemia time, min | 41 (36.3-48) | 39 (34-49) | 0.162 |  | 41.5 (35.8-49) | 39 (34-49) | 0.189 |
| Cold ischemia time, min | 97.5 (73.8-123.3) | 102 (65-126) | 0.892 |  | 96.5 (75-127.3) | 102 (65-126) | 0.739 |
| Transplant era |  |  | 0.507 |  |  |  | 0.872 |
| 2000-2009 | 73 (32.4%) | 27 (35.5%) |  |  | 27 (36.5%) | 27 (36.5%) |  |
| 2010-2019 | 128 (56.9%) | 38 (50.0%) |  |  | 39 (52.7%) | 37 (50.0%) |  |
| 2020-2023 | 24 (10.7%) | 11 (14.5%) |  |  | 8 (10.8%) | 10 (13.5%) |  |
| PBC, primary biliary cholangitis; PSC, primary sclerosing cholangitis; NASH, nonalcoholic steatohepatitis; MELD, model for end-stage liver disease; GRWR, graft-to-recipient weight ratio | | | | | | |  |
