# Supplementary material for: Association Between Distance to the Transplant Center and Survival Following Living Donor Liver Transplantation
Source: Ann Gastroenterol Surg. 2025 Jun 9;9(6):1322–33. doi: 10.1002/ags3.70051 (PMC12586949; doi:10.1002/ags3.70051)
Supplement: Supplementary file 3 — Table S2. Posttransplant complications in the matched cohort. [file AGS3-9-1322-s004.docx]

|  |  |  |  |
| --- | --- | --- | --- |
|  | **Gr 1 ＋Gr 2(n=74)** | **Gr 3 (n=74)** | **p-value** |
|  |  |  |  |
| Hepatic artery thrombosis, n (%) | 2 (2.7%) | 7 (9.5%) | 0.166 |
| Portal vein thrombosis, n (%) | 10 (13.5%) | 10 (13.5%) | 1 |
| Biliary complications, n (%) | 15 (20.3%) | 9 (12.2%) | 0.181 |
| Acute cellular rejection, n (%) | 26 (35.1%) | 23 (31.1%) | 0.600 |
| Early allograft dysfunction, n(%) | 21 (28.4%) | 27 (36.5%) | 0.292 |
| Small-for-size syndrome, n (%) | 18 (24.3%) | 21 (28.4%) | 0.608 |
|  |  |  |  |

**Supplementary Table 2. Posttransplant complications in the matched cohort**
