# Supplementary material for: Association Between Distance to the Transplant Center and Survival Following Living Donor Liver Transplantation
Source: Ann Gastroenterol Surg. 2025 Jun 9;9(6):1322–33. doi: 10.1002/ags3.70051 (PMC12586949; doi:10.1002/ags3.70051)
Supplement: Supplementary file 4 — Table S3. Graft function at the time of discharge. [file AGS3-9-1322-s005.docx]

| **Supplementary Table 3. Graft function at the time of discharge** | | | | |
| --- | --- | --- | --- | --- |
|  | **Gr 1** | **Gr 2** | **Gr 3** | ***P*** |
| AST | 25 (17-34) | 26 (18-41) | 29 (18-45) | 0.421 |
| ALT | 32 (19-48) | 29 (17-42) | 29 (16-70) | 0.553 |
| Bilirubin | 1 (0.7-1.6) | 0.9 (0.6-1.4) | 0.9 (0.6-1.4) | 0.116 |
| INR | 1.1 (1.0-1.2) | 1.1 (1.0-1.2) | 1.1 (1.0-1.2) | 0.415 |

AST, aspartate aminotransferase; ALT, alanine aminotransferase; INR, international normalized ratio
