# Supplementary material for: Association Between Distance to the Transplant Center and Survival Following Living Donor Liver Transplantation
Source: Ann Gastroenterol Surg. 2025 Jun 9;9(6):1322–33. doi: 10.1002/ags3.70051 (PMC12586949; doi:10.1002/ags3.70051)
Supplement: Supplementary file 5 — Table S4. Causes of death or graft failure and retransplant rate in the matched cohort. [file AGS3-9-1322-s001.docx]

|  |  |  |  |
| --- | --- | --- | --- |
|  | **Gr 1+ Gr 2 (n=74)** | **Gr 3 (n=74)** | ***P*** |
| Causes of death or graft failure |  |  |  |
| Primary graft failure | 1 (1.4%) | 4 (5.4%) | 0.366 |
| Infectious disease | 11 (14.9%) | 11 (14.9%) | 1 |
| PTLD or malignancy | 7 (9.5%) | 5 (6.8%) | 0.547 |
| Recurrence of primary disease | 2 (2.7%) | 2 (2.7%) | 1 |
| Vascular complications | 1 (1.4%) | 3 (4.1%) | 0.620 |
| Rejection | 0 (0%) | 2 (2.7%) | 0.497 |
| Retransplant rate | 8 (10.8%) | 1 (1.4%) | 0.034 |

**Supplementary Table 4. Causes of death or graft failure and retransplant rate in the matched cohort**
